# Linguistic and attentional factors – Not statistical regularities – Contribute to word-selective neural responses with FPVS-oddball paradigms^☆^

**DOI:** 10.1016/j.cortex.2024.01.007

**Published:** 2024-04

**Authors:** Aliette Lochy, Bruno Rossion, Matthew Lambon Ralph, Angélique Volfart, Olaf Hauk, Christine Schiltz

**Affiliations:** a Institute of Cognitive Science and Assessment, University of Luxembourg, Esch-sur-Alzette, Luxembourg; b Psychological Science Institute (IPSY), UCLouvain, Louvain-La-Neuve, Belgium; c Université de Lorraine, CNRS, Nancy, France; d CHRU-Nancy, Service de Neurologie, Nancy, France; e MRC Cognition and Brain Sciences Unit, University of Cambridge, UK; f School of Psychology and Counselling, Faculty of Health, Queensland University of Technology, Australia

**Keywords:** FPVS, Frequency-tagging, EEG, Visual word recognition, Statistical learning, Attention

## Abstract

Studies using frequency-tagging in electroencephalography (EEG) have dramatically increased in the past 10 years, in a variety of domains and populations. Here we used Fast Periodic Visual Stimulation (FPVS) combined with an oddball design to explore visual word recognition. Given the paradigm's high sensitivity, it is crucial for future basic research and clinical application to prove its robustness across variations of designs, stimulus types and tasks. This paradigm uses periodicity of brain responses to measure discrimination between two experimentally defined categories of stimuli presented periodically. EEG was recorded in 22 adults who viewed words inserted every 5 stimuli (at 2 Hz) within base stimuli presented at 10 Hz. Using two discrimination levels (deviant words among nonwords *or* pseudowords), we assessed the impact of relative frequency of item repetition (set size *or* item repetition controlled for deviant versus base stimuli), and of the orthogonal task (focused *or* deployed spatial attention). Word-selective occipito-temporal responses were robust at the individual level (significant in 95% of participants), left-lateralized, larger for the prelexical (nonwords) than lexical (pseudowords) contrast, and stronger with a deployed spatial attention task as compared to the typically used focused task. Importantly, amplitudes were not affected by item repetition. These results help understanding the factors influencing word-selective EEG responses and support the validity of FPVS-EEG oddball paradigms, as they confirm that word-selective responses are linguistic. Second, they show its robustness against design-related factors that could induce statistical (ir)regularities in item rate. They also confirm its high individual sensitivity and demonstrate how it can be optimized, using a deployed rather than focused attention task, to measure implicit word recognition processes in typical and atypical populations.

## 1 Introduction

In recent years, a frequency-tagging approach using electroencephalograhy (EEG) based on ‘oddball’, or ‘oddball-like’ design, has been successfully applied to explore visual recognition in the nonverbal (objects, faces, or numerosities; e.g., Guillaume et al., 2018, 2020; Hauk et al., 2021; Liu-Shuang et al., 2014; Marinova et al., 2021; Marlair et al., 2021; Nurdal et al., 2021; Retter et al., 2020; Stothart et al., 2017) and verbal domains (letters, words, word semantic categories; e.g., Lochy et al., 2015; Volfart et al., 2021). This fast periodic visual stimulation (FPVS) oddball approach is typically based on variable exemplars of a frequent stimulus category (or “base” category) presented at a rapid rate (e.g., 10 Hz), interrupted at a slower periodic rate (usually 1/5, e.g., 2 Hz) by a contrastive stimulus category (or “deviant” category) (Rossion et al., 2020). If the deviant category (e.g., words) is discriminated from the base category (e.g., non-words or pseudo-words, NW and PW hereafter), it elicits a neural response measurable in the frequency domain at exactly the frequency of the deviant category (e.g., 2 Hz) (Lochy et al., 2015). This approach has substantial advantages in terms of objectivity as responses occur at experimentally predefined frequencies. Furthermore, it is characterized by high sensitivity: because of its high signal-to-noise ratio (SNR), the neural response can be measured in various sensitive populations such as infants, children or patients (e.g., de Heering & Rossion, 2015; Lochy et al., 2016, 2018, 2019; Peykarjou et al., 2017; Vettori et al., 2019) in only a few minutes of recordings. Finally, neural responses are measured without requiring any explicit task, another advantage especially when collecting data in developmental or clinical populations.

Following the first study in French language (Lochy et al., 2015), the FPVS-EEG oddball approach was used to measure category-selectivity to visual letters and words. It has revealed left hemispheric cortical specialization for letters in young pre-readers related to their letter knowledge (Lochy et al., 2016; Wang et al., 2022), neural changes in beginning readers (van de Walle de Ghelcke et al., 2021; Wang et al., 2023), impact of teaching methods for learning to read (van de Walle de Ghelcke et al., 2020) and the neural basis of letter-selective and (pre)lexical responses in the left ventral occipito-temporal cortex with intracerebral recordings (Lochy et al., 2018). It has also recently been successfully extended to German (Aristei et al., 2017), though evidence of its suitability to English is currently lacking (Barnes et al., 2021; Wang et al., 2021).

Given FPVS-EEG's wide applicability, the main objective of the current study is to assess the validity and robustness of this approach in triggering word-selective neural responses. In particular, it is crucial to ascertain that the categorical responses measured with this paradigm are linguistic in nature (orthographic, lexical, or semantic), and do not stem from potential confounding peripheral processes or design factors. Specifically, using two levels of discrimination coarseness (words among nonwords – a coarse prelexical contrast – or words among pseudowords – a fine-grained lexical contrast), we explore how item repetition rate (implying potential statistical learning) modulates responses to words. A second aim is to explore if the type of orthogonal attention task, spatially focused or deployed, influences amplitude of responses. Finally, a last objective is to evaluate the sensitivity of this approach at the individual participant level.

Our first objective is to understand if word-selective neural responses elicited in this approach are truly linguistic (orthographic, lexical, or semantic). Indeed, responses to deviant words might well stem from relative differences in item repetition between base and deviant categories (de Rosa et al., 2022) rather than from linguistic processes, since in such oddball paradigms the rare and frequent sets of stimuli differ in the number of exemplar repetitions, or *relative item frequency*. That is, when two sets of an identical number of stimuli are used (e.g., 30 per set) and if the deviants are presented every five items (1/5), then the relative frequency of each *item* differs across categories (e.g., Lochy et al., 2015). Over a certain period, the base stimuli are presented 4 times more often than the deviants. Thus, with this paradigm, either the number of exemplars per set is matched (and item repetition is not, as in most previous studies) or the number of exemplar *repetitions* is matched (and the number of stimuli per set differs).

An important question is whether the relative item frequency can contribute, at least in part, to the measured neural discrimination response. That is, one cannot exclude that items of the deviant set would be discriminated, not because of their experimentally manipulated properties (e.g., the psycholinguistic contrast between words and non-words), but merely because they are repeated less often during the stimulation sequences. Indeed, seminal studies in language acquisition revealed fast learning of the statistical structure of meaningless syllable sequences varying in transitional probabilities in both adults and infants (Saffran, Aslin, et al., 1996; Saffran, Newport, et al., 1996). Since then, the sensitivity of our cognitive and neural system to detect structured patterns of the environment, also called *implicit statistical learning*, has been widely documented in a variety of domains (e.g., Arciuli & Simpson, 2012; Dienes et al., 1991; Frost et al., 2019; Kidd & Arciuli, 2016; Misyak & Christiansen, 2012; Misyak et al., 2010; Saffran, Aslin, et al., 1996; Saffran, Newport, et al., 1996; Shafto et al., 2012). Therefore, in FPVS-EEG studies, one cannot exclude that oddball responses to deviant stimuli are generated or modulated by the statistical regularities in the stimuli distribution and relative item frequency, and the patterned periodicity of occurrence of deviants among base stimuli, independently of their categorical (in this case linguistic) nature. Intriguingly, two recent FPVS-EEG studies suggested that it could be the case, showing that arbitrary groupings of digits (Guillaume et al., 2020) or of letter-strings (de Rosa et al., 2022) gave rise to some discrimination response, presumably due to the difference in relative item frequency, as there was no a priori reason for them to be discriminated from each other.

To investigate the potential impact of the relative exemplar frequency within base/deviant categories on neural responses to deviant words, we contrasted stimulation sequences in which set size was controlled, and the number of exemplar repetitions differed between base and deviant (i.e., 30 deviants -repeated 4 times each in a sequence, and 30 base stimuli repeated 16 times each in a sequence), to stimulation sequences in which the number of item repetitions, but not the set size, was controlled (i.e., 30 deviant and 120 base stimuli, all repeated 4 times in a sequence). Several hypotheses can be formulated. First, if implicit statistical learning about exemplar repetition is the only source of the neural oddball response, then discrimination of deviants should occur *only* when the set size is controlled (and item repetition rate is not). Such an observation would be in line with the significant discrimination occurring between sets that do not differ linguistically (de Rosa et al., 2022), and would imply that the linguistic status of words is not a key variable in FPVS-EEG responses, thus that these responses might have been wrongly interpreted in previous studies (Lochy et al., 2015, 2018; Volfart et al., 2021). Thus, *no response* should occur when item repetition rate is controlled, as there would be no difference in relative item frequency between words and base stimuli, as was observed for English words among pseudowords (Barnes et al., 2021). Alternatively, the neural oddball response to words may have *nothing* to do with statistical learning of regularities, so that discrimination of deviants would occur equally strongly in the two design types. Finally, *both* statistical learning and high-level word-selectivity could contribute to the neural discrimination. In that case, a quantitative difference between the two conditions should be observed, with discrimination responses being larger when set size is controlled (and item repetition differs), but still present when item repetition is controlled (and set size differs).

**Fig. 1:**
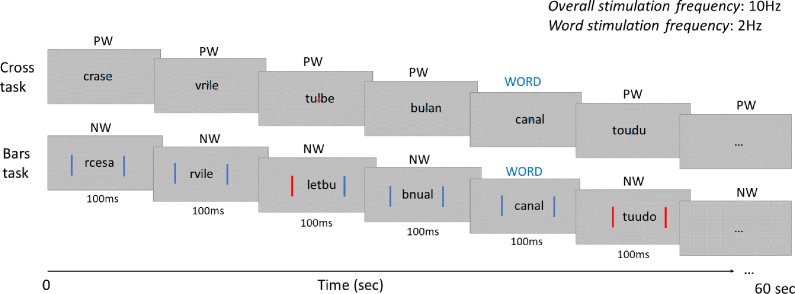
Experimental design. Sequences of letter-strings were presented at 10 Hz (10 stimuli/sec) during 60 sec. Deviant stimuli (words) were inserted every five items (at 2 Hz) among two types of base stimuli: pseudowords (PWW, top row) or nonwords (NWW, bottom row), inducing a fine-grained or a coarse discrimination contrast. Relative exemplar frequency of base and deviant stimuli was manipulated (*not represented in the Figure*), controlling either item repetition (with different set sizes for base and deviant stimuli), or set size (with different number of item repetitions for base and deviant stimuli). Two different orthogonal attentional tasks were implemented, requiring to monitor colour-changes either to a central cross (top row), or vertical bars (bottom row) flanked left and right of the letter-strings. Participants had to respond when the cross turned red, or when both bars turned red at the same time. Each type of attentional task (bars/cross) was performed on each discrimination type (NWW/PWW) and each control type (set size/exemplar repetition), resulting in a 2 × 2 × 2 design.

The second objective of this study was to assess the potential effect of the orthogonal attentional task performed during FPVS. In most previous FPVS-EEG studies with complex stimuli, attention is directed towards changes of colour to a fixation cross in the centre of the screen (since Rossion & Boremanse, 2011). This means that discrimination responses to deviant stimuli do not depend on explicit processing of these stimuli. However, spatial distribution attention may well allow better/worse processing of the stimuli. As used with letter strings, a central cross task might have primarily focused attention on the central/foveal letters (Nazir et al., 2004; Yeshurun & Carrasco, 1998) not located at the optimal fixation point for visual word recognition (*preferred landing position*, Rayner, 1998). Moreover, a fixation cross superimposed on letters partly masks 1 or 2 letters, depending on stimulus size (Fig. 1). Considering this issue, here we assess the impact of two different tasks requiring either focused or deployed spatial attention. The latter was implemented by colour-monitoring of two simultaneous vertical bars left and right from the letter string [see Fig. 1; e.g., as used recently in Yan et al., (2022) with face stimuli]. We expect that this alternative task might induce processing of a larger portion of the visual field and thus increase attention to all letters presented, as suggested by studies showing that the size of attentional focus is adjusted to the size of the spatial cue (Eriksen & St James, 1986; Turatto et al., 2000) and may improve perceptual identification (Posner, 1988). Furthermore, some findings suggest that the impact of broadening attention by cueing both sides of the target might have a differential impact depending on lexicality of targets, being more beneficial for frequent words than pseudowords (Montani et al., 2014); although see Ducrot & Grainger (2007), who suggest no effect of spatial cueing on centrally presented targets. If this is the case even when no explicit identification is required, we expect the deployed attention task to give rise to larger word-selective responses than the focused task. Furthermore, this increase of responses should be selective to discrimination (because of enhanced processing) and should not influence general visual responses at the base stimulation rate.

Our third and final objective was to evaluate the sensitivity of the paradigm to elicit responses at the individual participant-level. In the princeps study of (Lochy et al., 2015), 8 participants out of 10 showed a significant response to words among nonwords or pseudowords. This was recently challenged in another, less orthographically transparent language (English) where only 10% of datasets were significant (10 participants, Barnes et al., 2021). Therefore, we examined this issue again on a larger sample of participants and different set of stimuli, as well as separately for each discrimination level (words in nonwords/pseudowords).

## 2 Material and methods

We report how we determined our sample size, all data exclusions, all inclusion/exclusion criteria, whether inclusion/exclusion criteria were established prior to data analysis, all manipulations, and all measures in the study.

### 2.1 Participants

Twenty-four adults with normal/corrected-to-normal vision were tested after they gave their written informed consent for a study approved by the Ethical Committee of the University of Luxembourg as conform to the Declaration of Helsinki. This sample size was determined a priori, based on studies using the same approach that used typically samples of 10–20 participants (Barnes et al., 2021; Liu-Shuang et al., 2014; Lochy et al., 2015; Stothart et al., 2017). Participants were neither aware of the goal of the experiment, nor that a change of stimulus type occurred at a periodic rate during stimulation. Two participants were excluded because their EEG data was too noisy and thus analyses were performed on 22 participants attending University (a priori inclusion criteria: native French-speakers, right-handed, half males/females, age 18–35 years old; a priori exclusion criteria: reading difficulty or diagnosed dyslexia; current sample: 11 males, mean age = 23.54 years; range = 20–29 years).

### 2.2 EEG testing stimuli

Thirty French words (concrete frequent nouns) were chosen (Supp. Mat Table S1). All were 5 letters long and presented in their singular form. Lexical frequency [Celex database in WordGen (Duyck et al., 2004)] was on average 86.77 per million (min: 13.42, max: 734.51, SD.: 129.90). 17 words were monosyllabic, 12 bi-syllabic and 1 contained three syllables. For the fine-grained lexical discrimination level, four different sets of 30 pseudowords were created to match the words pairwise in consonant–vowel structure, number of syllables, number of orthographic neighbours and bigram frequency [all stimuli are available in Supp. Mat. As an example, for the word “radio” (CVCVV), PW1: “virie”, PW2: “pomue”, PW3: “bario”, PW4: “tovui”]. Each set of PW did not differ from words on these variables (see Table 1) and did not differ from each other. For the coarse-grained pre-lexical discrimination level, four different sets of nonwords were also created. To do so, letters constituting each PW were shuffled to give rise to a (mostly) non-pronounceable item, matched in letter identity only with PW (to avoid letter frequency differences between conditions) and purposely not matched with words in number of orthographic neighbours or bigram frequency (for the examples provided above: NW1: “verii”, NW2: “mpeue”, NW3: “rbaio”, NW4: “vtoiu”). The characteristics of these 4 sets of 30 non-words are described in Table 1. Each set of NW did not differ from each other on these two variables (stimuli in Supp. Mat). In total, there were thus 30 words, 120 PW and 120 NW.

**Table 1:** Stimulus characteristics.

Set type	Number of orthographic neighbours					Bigram frequency				
	Mean	*t-test (words)*	Min	Max	SD	Mean	*t-test (words)*	Min	Max	SD
**Words**	**2.63**		**0**	**8**	**2.02**	**11,627.3**		**3170**	**17,911**	**3736.6**
PW1	2.65	*p = .996*	0	8	2.04	11,615.3	*p = .873*	3614	17,598	3323.4
PW2	2.60	*p = .937*	0	8	2.06	11,634.6	*p = .980*	3405	17,471	3225.6
PW3	2.60	*p = .952*	0	8	2.17	11,610.6	*p = .982*	3629	17,397	3812.9
PW4	2.60	*p = .977*	0	8	2.55	11,623.9	*p = .969*	3892	18,627	3331.9
NW1	.10	*p < .000*	0	1	.40	5764.3	*p < .000*	2129	16,287	3371.1
NW2	.03	*p < .000*	0	1	.18	7255.3	*p < .000*	1058	17,399	4459.8
NW3	.10	*p < .000*	0	1	.31	7018.8	*p < .000*	880	15,554	3886.6
NW4	.13	*p < .000*	0	2	.35	5930.6	*p < .000*	1135	15,511	3274.1

*Note*: The table depicts mean, min, max and standard deviation for the number of orthographic neighbours (first column) and for bigram frequency (second column) for words and each set of pseudowords (PW) and nonwords (NW). The *p*-values of *t*-tests comparing each list of PW and NW to words show that PW did not differ, while NW differed on these two parameters from words.

Final stimuli were maximum 238 × 71 pixels. At a distance of 1 m, displayed with a 1920 × 1080 pixels resolution, they had an average size of 3.97 × 1.26 degrees of visual angle. Stimuli were presented at the centre of the screen with no immediate repetition of the same stimulus.

### 2.3 Procedure

Each stimulation sequence started with the fixation corresponding to the task (cross or vertical bars) for 2–5 sec, 2 sec of gradual stimulation fade in, 60 sec of the stimulation sequence, and 2 sec of gradual fade out (see Fig. 1). As in previous studies (e.g., Lochy et al., 2015), stimuli were presented by means of sinusoidal contrast modulation at a base frequency rate of 10 Hz (i.e., one item every 100 msec) (Fig. 1). Stimuli were presented using custom in-house built software running under Java SE Version 8. Every sequence had the same structure: stimuli of the base category were presented at 10 Hz (non-words or pseudo-words), and every fifth item was a deviant stimulus (words, frequency of 2 Hz, thus every 500 msec).

Four conditions were presented, in a 2x2 design crossing Discrimination Level (coarse/fine) and Control Type (set size controlled/item repetition controlled). In the *coarse discrimination level*, words were presented every five items among strings of non-words (NWW); while in the *fine-grained discrimination level*, words were presented every five items among strings of pseudowords (PWW). Since the response to the deviant stimuli in the FPVS-oddball paradigm is not measured in absolute terms, but as an index of a *differential* processing between base and deviant stimuli (i.e., all common processes between base and deviant stimuli project to the common base rate response, which can be also objectively identified in the EEG spectrum; see Rossion et al., 2020 for review), we expected to replicate the finding that words among NW trigger a larger amplitude response than words among PW, using a different stimulus set than the princeps study (Lochy et al., 2015) and a larger sample of participants (N = 10 *vs* 22). The larger response in the coarser contrast may indeed be based on pre-lexical orthographic plausibility detection of regularities in the combination of co-occurring letters coded at the neural level (Bouhali et al., 2014, 2019; Dehaene et al., 2005; Grainger & van Heuven, 2003; Mariol et al., 2008; Vinckier et al., 2007). The PWW condition controls these pre-lexical factors, supporting the interpretation of responses as stemming from lexical levels (whole word-forms are recognized) or semantic levels (only words have a meaning). *Set size* was controlled when, as per the original study (Lochy et al., 2015), 30 words were presented as deviants among 30 base stimuli (set 1 of nonwords or set 1 of pseudowords). However, in that case, the number of repetitions is much higher for the base stimuli: at 10 Hz, 600 items are displayed in a 60-sec sequence, among which 480 base stimuli (30 PW or NW × 16) and 120 deviants (30 W × 4). *Item repetition* was controlled when 30 words were presented among 120 different base stimuli (either nonwords or pseudowords). In that case, each item was presented an identical number of times (4×) during each 60-sec sequence, but the size of sets differed.

Two orthogonal tasks were designed to assess the impact of focus or deployed spatial attention (Fig. 1). The focused attention required to monitor a colour change occurring randomly on a cross in the centre of the screen, while stimuli were displayed, by pressing the space bar upon each colour-change (blue-to-red). The deployed attention required to monitor simultaneously two vertical bars displayed left and right of the stimuli (−.3 and .3 in unit coordinates), where colour changes could occur either on the left, on the right, or on both at the same time, and to press the space bar when both vertical bars changed colour simultaneously. This task was chosen to ensure that participants would keep deploying attention on both sides, and not use a strategy looking at the left or right bar only, which would draw attention away from the centred stimulation sequence. In each task, there were 8 colour-changes per 60 sec sequence, that occurred randomly and lasted 200 msec each. While for the cross, all colour-changes required a key press (“go” trials), for the bars, approximately half of the trials required a key press (“go” trials; 4.05/8) and half did not (“no-go” trials; 3.95/8). All stimuli were similar between the two tasks. These two tasks were presented by block and their order was counterbalanced across participants.

Each sequence was repeated twice in each task, for a total of 4 conditions × 3 repetitions × 60 sec per task. A pause was taken between each of the 24 sequences, which were initiated manually to ensure low-artefact EEG signals.

### 2.4 EEG acquisition and preprocessing

Participants were seated comfortably at 1 m from the computer screen in a quiet room of the University. EEG was acquired at 1024 Hz using a 68-channel Biosemi Active II system (Biosemi, Amsterdam, Netherlands), with electrodes including 64 channels standard 10–20 system locations (http://www.biosemi.com) plus a row of posterior electrodes including PO9, I1, I2, PO10. The magnitude of the offset of all electrodes, referenced to the common mode sense (CMS), was held below 50 mV. EEG analyses were carried out using Letswave 6 (https://github.com/NOCIONS/letswave6), and Matlab 2012 (The Mathworks). After FFT band-pass filtering between .1 and 100 Hz, EEG data were segmented to include 2 sec before and after each sequence, resulting in 64-sec segments (−2 to 62 sec). Data files were then resampled to 512 Hz to reduce file size and data processing time. Artefact-ridden or noisy channels were replaced using linear interpolation (on average, 1.54% of channels). All channels were re-referenced to the common average. EEG recordings were then segmented again from stimulation onset until 59.998 sec, corresponding to the largest number of complete cycles of 500 msec (120 cycles) at the 2 Hz frequency within the 60 sec of stimulation period.

The data were analysed following the same procedure as in previous studies with similar paradigms (e.g., Liu-Shuang et al., 2014; Lochy et al., 2015, 2016; Retter & Rossion, 2016; Rossion et al., 2012) but is nevertheless detailed here.

### 2.5 Frequency domain analysis

Per condition, the three stimulation sequences were averaged in the time domain for each participant, to increase SNR. A Fast Fourier Transform (FFT) was applied to the averaged time-window, and normalized amplitude spectra were extracted for all channels. This yielded EEG spectra with a high frequency resolution (1/59.998 sec = .016 Hz), increasing SNR and allowing unambiguous identification of the response at the exact frequencies of interest (i.e., 10 Hz for the base stimulation rate and 2 Hz and harmonics for the other category stimulation). Given that our response of interest falls into a strictly defined frequency bin (related to stimulation frequency), we considered surrounding bins as noise, or baseline. The latter is defined as the 20 surrounding bins of each target bin, excluding the immediately adjacent and the extreme (min and max) bins (Liu-Shuang et al., 2014; Rossion et al., 2012; Srinivasan et al., 1999). We then computed three indices. First, the signal-to-noise ratio (SNR) was estimated across the EEG spectrum, by dividing the amplitude at each frequency bin by the average amplitude of 20 surrounding bins (10 on each side; Liu-Shuang et al., 2014). Second, to quantify the responses of interest in microvolts, the average voltage amplitude of the 20 surrounding bins (i.e., the noise) was subtracted out (Retter & Rossion, 2016). This is done because the amplitude at any given frequency is considered as a combination of signal and noise (Heinrich et al., 2009). Finally, Z-scores were computed based on the grand-averaged amplitude spectrum for each condition, to assess the significance of the response at each stimulation frequency (base-10 Hz, categorical change-2 Hz) and harmonics (Liu-Shuang et al., 2014; Lochy et al., 2015). Z-scores [*Z(x) = x-mean(noise)/SD (noise*)] were considered significant if larger than 3.1 (*p* < .001, one-tailed, signal > noise).

In order to quantify the periodic response distributed across several harmonics, the baseline subtracted amplitudes of significant harmonics (excluding the base stimulation frequency) were summed for each participant, task, and condition (Retter & Rossion, 2016).

No part of the study procedures or analysis plans was preregistered prior to the research being conducted. All study data and analysis code are available on the Open Science Framework platform (https://osf.io/kvfgx/). The complete list of stimuli is provided in Supp. Mat.

## 3 Results

### 3.1 Behavioural colour-change task

We first analysed accuracy and response times in the two tasks with paired *t*-tests. Due to recording failures, the data of 3 participants were lost. Behavioural analyses were thus performed on 19 participants.

There was a non-significant trend for higher accuracy (hit rate) in the bars task (96.9%) than in the cross task (95.23%) [t(18) = 1.999; *p* = .061]. However, false alarms were higher in the bars (5.04%) than in the cross task (3.02%) [t(18) = 2.648; *p* = .016], which could be expected as only the bars task contained No-Go trials (e.g., colour-change on one side only). Finally, correct hits RT were longer for the bars (501.3 msec) than the cross task (462.6 msec) [t(18) = 3.065; *p* = .007]. In the bars task, there was a non-significant trend for RT on false alarms (No-Go trials) to be shorter than RT on Go trials (457.1 msec *vs* 501.3 msec) [t(18) = 1.532; *p* = .143]. In sum, these results indicate that both tasks were very well performed by the participants, with the bars task being slightly more demanding than the cross task.

### 3.2 Categorical discrimination responses

**Fig. 2:**
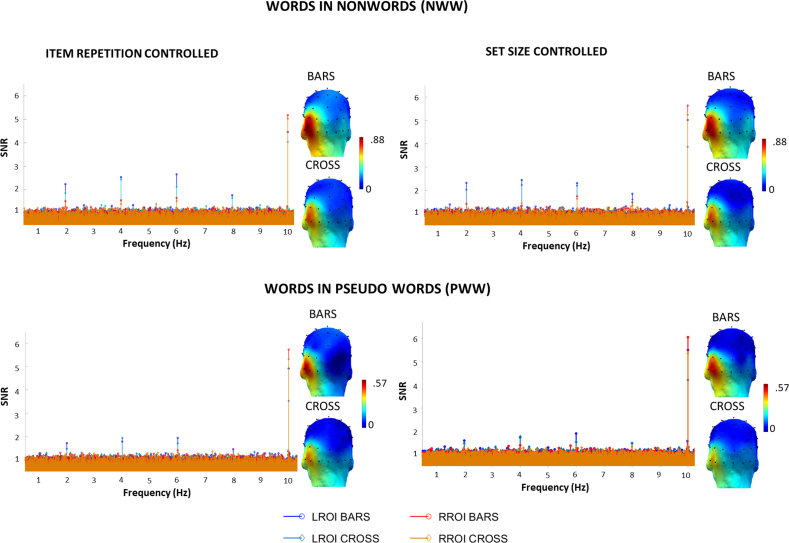
EEG spectra (SNR, baseline level = 1) and scalp topographies (scale in μV) of categorical discrimination responses for words (i.e., word-selective neural responses) among non-words (top row) or pseudowords (bottom row). The SNR is shown in left and right regions-of-interest (LROI/RROI) and for each task (bars/cross). It reveals in each condition, task, and control type, clear discrimination responses for words at 2 Hz and harmonics (significant up to 8 Hz). The peak at 10 Hz represents the base rate (general visual) response. While the type of control (set size or item repetition) did not influence response amplitudes, the orthogonal task induced stronger word discrimination responses when participants monitored two lateral vertical bars as a central cross.

Across tasks and conditions, averaged Z-scores were significant (*z* > 3.1, *p* < .001) for discrimination responses from 2 Hz up to 8 Hz (i.e., 4 harmonics). Scalp topographies of the sum of harmonics show a larger word-selective response in the left hemisphere (Fig. 2), as in previous studies (de Rosa et al., 2022; Lochy et al., 2015). Based on this, we selected a priori electrodes of interest in the LH (including 5 electrodes around P07) as well as their contralateral homologues to define LH and RH regions-of-interest (ROI) for further analysis.

The sum of baseline corrected amplitudes for the four significant harmonics were submitted to a 2 (*Tasks*: cross versus bars) × 2 (*Discrimination level*: fine versus coarse-grained) × 2 (*Control Type*: set size versus item repetition) × 2 (*Hemisphere*s: LH versus RH) repeated-measures ANOVA.

**Fig. 3:**
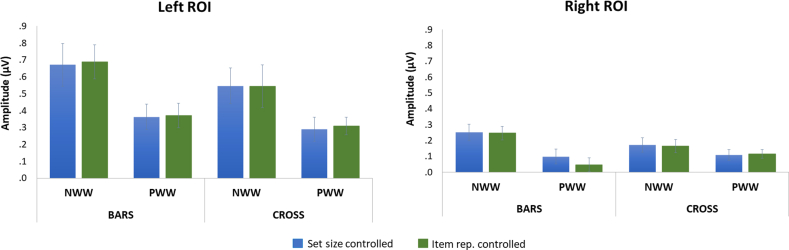
Word-selective EEG amplitudes (in μV, sum of harmonics), as a function of control type (blue: controlled set size; green: controlled item repetition), discrimination level (words among non-words: NWW; words among pseudowords: PWW) and task (bars, cross). The left panel represents the left ROI and the right panel the right ROI.

There was a main effect of *Hemisphere*, *F*(1,21) = 24.219; *p* < .0001; *BF* = 1192.193, with (about three times) larger responses in the LH (.473 μV) than in the RH (.151 μV) (Fig. 2, Fig. 3). *Tasks* also significantly influenced response amplitudes, *F*(1,21) = 5.206; *p* < .033; *BF* = 9.173, with larger EEG amplitudes in the bars (.342 μV) than in the cross task (.282 μV). There was a main effect of *Discrimination level F*(1,21) = 22.092; *p* < .0001; *BF* = 530.632, with stronger responses when words were inserted in NW (.411 μV) than in PW (.213 μV). In contrast, the factor *Control Type* was not significant F(1,21) < 1; *p* = .94; item repetition controlled: .311 μV; set size controlled: .313 μV; *BF* = .219.

There were also several significant interactions modulating these effects that were further explored with pairwise comparisons using Bonferroni correction. First, the interaction *Hemisphere* × *Task F*(1,21) = 7.266; *p* < .014 was due to an effect of *Task* in the LH (*p* < .011) (cross: .42 μV; bars: .52 μV) but not in the RH (cross: .16; bars: .14 μV). Second, there was an interaction between *Hemisphere* and *Discrimination level, F*(1,21) = 6.456; *p* < .019. Responses to words among NW (NWW) were higher than those among PW in each hemisphere (in the LH; *p* < .001; in the RH; *p* < .004), but the difference was stronger in the LH (.279 μV) than in the RH (.116 μV). The left dominance in response amplitude was present in each discrimination level as shown by contrasts of hemispheres (for NWW, *p* < .001; and for PWW, *p* < .001). Finally, there was a trend for an interaction between *Task* and *Discrimination Level F*(1,21) = 4.196; *p* = .053, with the effect of task being marginally stronger for NWW sequences (bars: .465 μV, cross: .357 μV) than for PWW sequences (bars: .220 μV, cross: .206 μV). All other interactions were not significant (Fs < 1).

### 3.3 Base rate responses

Responses at the base rate (10 Hz) were significant up to 4 harmonics (40 Hz), when averaging across tasks and conditions, with the largest EEG amplitudes on posterior channels of the occipital medial region, as in previous studies (e.g., Lochy et al., 2015) (ranked as O2, Oz, Iz and O1 for the 4 first channels). We defined a medial-occipital region (MO-ROI) including Oz, Iz, O1 and O2 and we analysed the effect of *Tasks* (cross versus bars) × *Discrimination level* (fine versus coarse-grained) × *Control Type* (item repetition versus set size) in a 2 × 2 × 2 repeated-measures ANOVA.

**Fig. 4:**
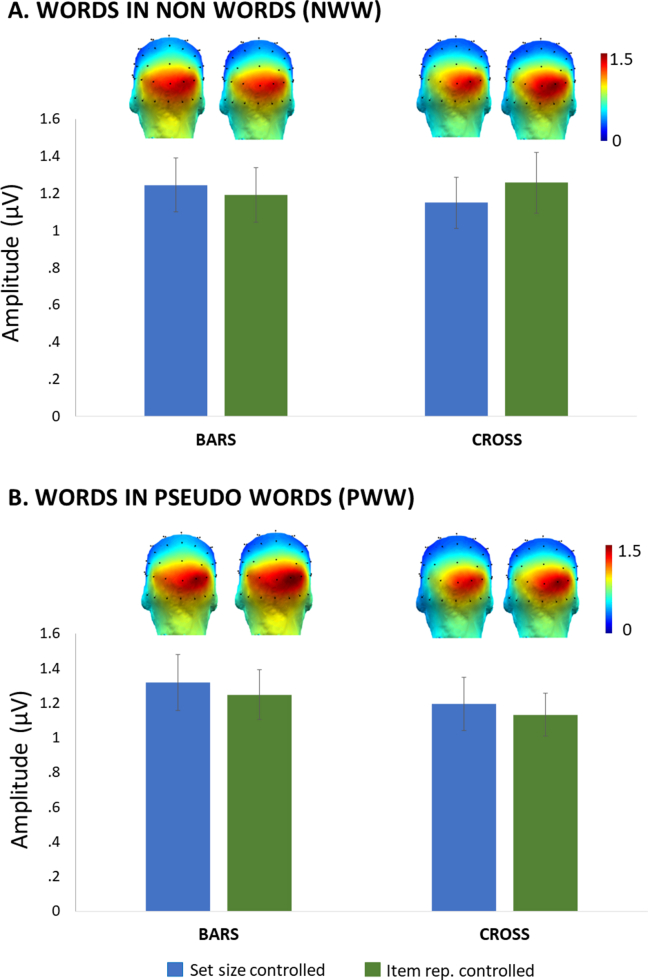
Base rate response amplitudes (in μV) at a medial-occipital region of interest, as a function of *Discrimination Level* (**A.** words in nonwords, NWW; **B.** words in pseudowords, PWW), *Task* (cross versus bars) and *Control Type* (item repetition versus set size).

There were no significant main effects (all Fs < 1) or interactions [*Task* × *Discrimination Level*, F(1,21) = 2.472; *p* = .131; *Task* × *Item repetition* F(1,21) = 1.329; *p* = .262; *Discrimination Level* × *Item repetition* F(1,21) = 1.140; *p* = .298; *Task* × *Item repetition* × *Discrimination Level* F(1,21) = 1.212; *p* = .283], suggesting that the overall general visual response did not differ across all these sequences showing strings of letters at 10 Hz, whatever the task or nature of the contrast (Fig. 4).

### 3.4 Individual level analysis

Given the highly significant effects obtained when analysing data at the group-level, we also assessed whether the approach is powerful enough to evidence clear responses at the individual level (which would also be critical to know for potential clinical application with individual patients). For this, individual Z-scores were computed for each participant, discrimination level, control type, and task, in each ROI, on non-corrected summed amplitudes for significant harmonics as determined at the group level (see Supplementary Fig. S1 for display of individual Z-scores and Fig. S2 with details in each cell of the design.).

Impressively, all of the 22 individual participants had a significant EEG response (*z* > 1.64) for words among non-words in *at least* one ROI, task, and control type. 15 out of 22 (68%) had a significant response *in all conditions* (independently of ROI), and no individual showed no response at all. For the fine discrimination level, i.e., words among pseudowords, 20 out of 22 participants showed a significant response in *at least* one ROI, task, and control type. Among them, 10/22 (45%) showed a significant response *in all conditions*, and only two individuals did not show any significant response.

**Fig. 5:**
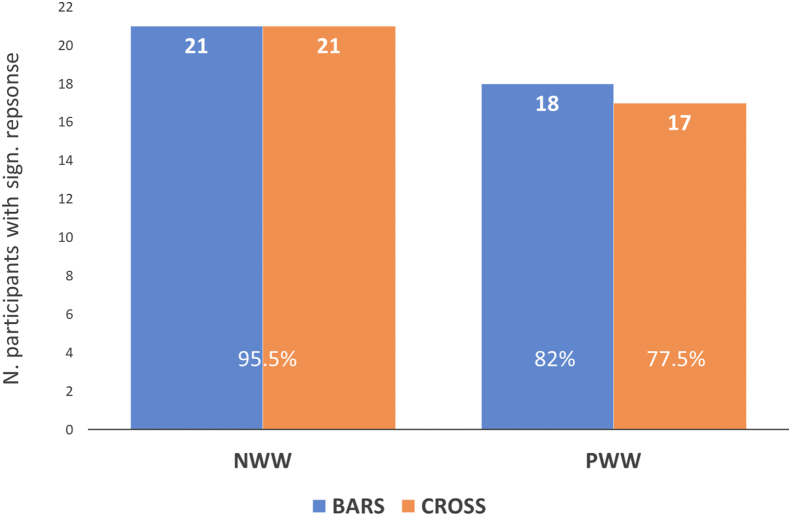
Number of participants (out of 22) showing a significant response in the left ROI per *discrimination coarseness condition* (NWW, PWW) and *task* (Bars, in blue and Cross in orange on the graph), in at least one of the *Control Types* conditions (item repetition or set size controlled).

Considering the left ROI only, as most of the word-selective response concentrates in that region, and not taking into account the *Control Type* factor (as it does not influence responses at the group level), the sensitivity of the paradigm is extremely good as can be seen on Fig. 5. For NWW, we were able to measure a significant response (set size or item repetition controlled) in 95% of participants, and for PWW, in 82% in the most sensitive bars task.

We also computed the correlation across individuals between conditions (bars versus cross, item number versus repetition controlled; PW versus NW) after calculating for each participant the average amplitude response in the LROI in each of the variables of interest.

**Fig. 6:**
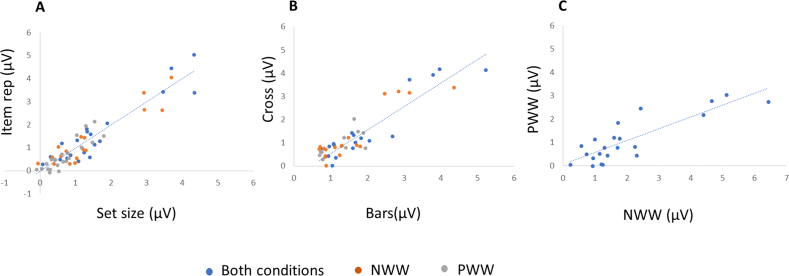
Scatter plots for individual word-selective responses illustrating correlations between: A. set size versus item repetition controlled; B. cross versus bars tasks; C. words in Pseudowords versus words in nonwords discrimination levels. In A & B, correlations were computed for both PWW and NWW conditions averaged, but individual data are also plotted for each condition separately (orange dots: NWW; grey dots: PWW).

Correlations were significant and high (Fig. 6) between the two *Control Types* (item repetition versus set size: Spearman Rho = .810; *p* < .001); between cross and bars tasks (Rho = .816; *p* < .001); and slightly lower between the two discrimination levels (Rho = .740; *p* < .001).

Finally, given that the increase of discrimination responses with deployed attention could be due to a general arousal effect due to the bars task being slightly more demanding, we correlated the amplitude increase between cross and bars tasks (averaging Conditions and Control Types), to the RT increase between these two tasks. These variables did not correlate, considering only the left ROI (Spearman Rho = .091; *p* = .729), or both ROIs together (Spearman Rho = .23; *p* = .37).

## 4 Discussion

Given the development and increasing use of an FPVS-oddball approach to study written letter and word-selective representations (Barnes et al., 2021; de Rosa et al., 2022; Lochy et al., 2015, 2016, 2018; Lochy & Schiltz, 2019; Pescuma et al., 2022; van de Walle de Ghelcke et al., 2020, 2021; Wang et al., 2021, 2022, 2023), the crucial aim of the present study was to ascertain that the processes that trigger word-selective responses in this paradigm are linguistic, and are not a side-effect of design factors that may induce statistical learning. Given that relative item frequency did not impact oddball responses, our findings strongly support the interpretation of FPVS word-selective responses as stemming from psycholinguistic processing of words. Second, we show for the first time that the paradigm can be further improved by using a deployed attentional task, presumably enhancing the unintentional processing of words. Finally, strengthening previous observations (Lochy et al., 2015), we found that the coarser discrimination contrast (prelexical) gives rise to the largest responses, but also that lexical responses are measurable at the individual participant level. These findings are discussed below.

### 4.1 Different levels of word-selectivity

With new stimuli and a larger sample of participants, we replicated a modulation of response amplitude as a function of discrimination coarseness, implemented by manipulating the wordlikeness of base stimuli (Lochy et al., 2015): word-selective responses were significantly larger when contrasted to non-words than pseudowords, and this especially in the left hemisphere.

This finding parallels list context influences observed in behavioural performance (Evans et al., 2012; Lupker & Pexman, 2010). In lexical decision tasks, the time to recognize a word is influenced by the wordlikeness of the list. This finding has been explained by a modulation of the word recognition threshold which is directly dependent on the overall level of activation in the lexicon (Lupker & Pexman, 2010). When the nonwords have very few orthographic neighbours (e.g., here in nonwords sequences), then the amount of activation is low, and words are recognized at modest thresholds (faster decision times) (Grainger & Jacobs, 1996). The opposite is true for nonwords that have several orthographic neighbours (here, in pseudowords sequences). Given that our task did not require any decision about lexicality, results suggest that these effects stem from pre-decisional processing stages. The simple viewing of words in different wordlikeness contexts was sufficient to trigger these effects, which supports the proposal that some word processing at the lexical/semantic level occurs even without explicit attention to any linguistic aspect of the stimulus (Brown et al., 2002), but processing can still be modulated by task (here, widening the size of the attention lens) (Chen et al., 2015; Strijkers et al., 2015).

Regardless of the level of contrast, the left occipito-temporal scalp topography of word-selective activity is in agreement with the known brain regions implicated in reading and visual word recognition, such as the left VOTC (ventral occipito-temporal cortex) including the so-called visual word form area (VWFA) (Cattinelli et al., 2013; Dehaene et al., 2005; Schurz et al., 2014; Wandell & Le, 2017). They also align with the word-selective intracerebral responses disclosed with this paradigm in the lateral portion of the left middle fusiform gyrus/occipito-temporal sulcus (Lochy et al., 2018). Two different routes for processing letter-strings have been proposed in the brain as a dorsal and a ventral stream in the left hemisphere (Cohen et al., 2008; Jobard et al., 2003; Levy et al., 2009; Pugh et al., 2001; Yeatman & White, 2021). The dorsal route starts from the posterior part of the VWFA and is connected to the parietal lobe (PL) involved in visual attention and serial processing (White et al., 2019; Yeatman et al., 2014). It further involves the superior temporal gyrus (STG) and posterior inferior frontal gyrus (IFG) and presumably subserves orthographic-to-phonological conversion (Cohen & Dehaene, 2009); thus it is activated when reading pseudowords (Levy et al., 2009). The ventral route involves the anterior part of the VWFA, related through the arcuate fasciculus to the anterior temporal lobe (ATL) and the anterior IFG (Pugh et al., 2001; Schlaggar & McCandliss, 2007; Yeatman & White, 2021). It is sensitive to words (by opposition to PW) and to their linguistic properties (Glezer et al., 2009, 2015; Hirshorn et al., 2016; Riesenhuber & Glezer, 2017). The responses that we observe for words among PW or NW could thus stem from different routes being activated by stimulus type, both PW and NW triggering non-lexical processes relying more on the dorsal stream, while words rely more on the ventral stream (Cohen & Dehaene, 2009).

The fact that the index of differential processing measured by this paradigm is lower for PWW than NWW sequences without changes of topography suggests that both fine-tuning to whole-words (e.g., lexical representations) and broad-tuning to plausible letter-strings (e.g., prelexical representations) are located in overlapping neural populations in the left fusiform gyrus (Glezer et al., 2009; Lochy et al., 2018). This could be further addressed in future studies using combined EEG-MEG and source estimation (e.g., Hauk et al., 2021).

### 4.2 Statistical regularities do not contribute to word-selective responses

The FPVS-oddball paradigm is very sensitive for capturing neural discrimination between a base and deviant categories of stimuli (for instance words as deviants among non-words or pseudowords as done here). It does, however, contain an inherent imbalance in exemplar repetition when, as often the case in such studies, set sizes of the two contrasted categories are equated. Given the importance of implicit statistical learning as a basic mechanism for extracting patterned regularities in the environment (Armstrong et al., 2017; Aslin, 2017; Christiansen, 2019; Frost et al., 2019), one could legitimately wonder if word-selective neural responses in previous studies related to their psycholinguistic characteristics actually reflected the detection of different frequency-of-occurrences of base and deviant stimuli. Thus, it was crucial to evaluate if, and how much of, the discrimination response for words in the FPVS-oddball approach is at least partly due to detection of item repetition differences rather than detection of words as lexical items with specific properties by comparison to base stimuli.

If there were cumulative effects of statistical learning about regularities and orthographic/lexical processes, responses should have been larger when item repetition was imbalanced (set size controlled). However, contrary to this prediction, our results show that word-selective responses did not differ in amplitude or lateralization whether set size or item repetition were controlled. One could argue that our sample was not sufficiently large to detect a small effect between the two types of controls. Yet, power analysis using Gpower (Faul et al., 2007) indicated that to detect *large* effects (f = .8), with 80% power using an F-test between repeated measures with an alpha of .05, a sample of 6 participants would be sufficient. For *moderate* (f = .5) or *small* (f = .2) effects, samples of respectively 10 and 52 participants would be recommended. However, the observed effect size was not even small here, but null [F = .005; partial η2 = .000, observed power (1-β) = .050]. Furthermore, the Bayes factor of .219 can be viewed as reasonable evidence that there is no difference between these two conditions. In addition, the correlation across individuals between these two conditions was very high (rho = .81), arguing for qualitatively similar processes underlying the word-selective response in both cases. In sum, there was no effect that could be attributed to statistical learning about exemplar repetition when using a deviant set of 30 items being presented less often than the base set of 30 items.

These observations are consistent with studies using this oddball-like paradigm in other domains. For instance, when contrasting (deviant) faces to (base) objects, the number of repetitions for exemplars in each category does not matter (Retter & Rossion, 2016). In this latter study, using a set of 46 faces and 248 objects images, the authors showed that the periodicity of deviant faces in 120 sec sequences presented at 12.5 Hz, from 1 face every 5 objects to 1 every 11 objects (ratios face/objects: 1/5, 1/7, 1/9 and 1/11) had no influence whatsoever on the amplitude and spatial localization of the face-selective response. In another study, faces were presented at a fixed 1 Hz rate in sequences varying in overall stimulation frequency, from 20 Hz to 3 Hz (Retter et al., 2020). In that case, the number of objects between face-deviants varied accordingly, i.e., faces were separated by 2 objects (3 Hz), or 5 (6 Hz), 11 (12 Hz) or even 19 (20 Hz). Again, the number of repetitions between deviants did not show any effect on amplitude of the face-selective response (Retter et al., 2020).

In contrast to these studies on faces and the present study on written words, we note that recent studies that found effects of statistical regularities, either by arbitrary grouping digits (Guillaume et al., 2020), or letters/characters-strings (de Rosa et al., 2022) used rather small sets of 4 or 8 items. Since statistical regularities must be easier to detect when the exact small set of stimuli is repeated, we think this factor could be key in statistical learning of novel groupings. If this is correct, future work should identify how many different items should be used to avoid these statistical learning effects. Since there are areas of research in which it is not possible to use many different items [e.g., in numerical cognition, when using Arabic digits as stimuli, there are maximum 10 elements (1–9, and 0)], future studies with FPVS-oddball like paradigms should be particularly careful, using changes in surface features of the stimuli to reduce/eliminate mere statistical regularity effects (see e.g., Marlair et al., 2022).

Interestingly, while we replicated the LH dominance in the scalp topography of word-selective responses, the study of de Rosa et al. (2022), identifying statistical learning effects with character strings found rather different scalp topographies: these responses were bilateral at posterior sites, without any effect of hemisphere. Furthermore, responses did not differ between conditions (words, pseudowords, non-words or artificial script), even though several more clusters were found for words. This posterior topography fits well with the visual networks that are activated by statistical learning tasks constituted of visual stimuli (Gavornik & Bear, 2014; Kok et al., 2012; Richter & de Lange, 2019). In contrast, our observation of a clear LH dominance is yet another argument in favour of interpreting the origin of the discrimination effect for words among NW or PW as being orthographic or lexical, rather than a mere detection of imbalance in item repetition.

### 4.3 Deployed versus focal attention

We found a relatively large effect of the attentional task in the left hemisphere, with larger word-selective responses in the deployed attention task than the focused attention task. The automatic word processing occurring in FPVS-EEG studies can therefore be modulated by an attentional manipulation, the same way Stroop effects are reduced by focussing spatial attention on a single letter of the word (e.g., Lachter et al., 2008; Stolz & Besner, 1999). The attention literature in general shows that too much focused attention may not be optimal, and that better performance in perceptual identification is obtained with an adequate allocation of attentional resources (Yeshurun & Carrasco, 1998). In written word recognition, a whole field of research investigated the effects of spatial cueing, assessing the consequences of processing words at an attended/unattended location, evaluating the spontaneous asymmetry of attention distribution to one or the other visual field, testing whether word processing requires attention or is immune to attentional orientation (Ramamurthy et al., 2021; Stein, 2014; White et al., 2019), or showing evidence for a role of visual spatial attention in prereaders to predicting future reading acquisition (Franceschini et al., 2012). Also, effects of spatial cueing on different types of written strings were investigated, reasoning that only the phonological route (hence, pseudoword identification) requires parsing into small units and therefore focused spatial attention (Ans et al., 1998; Perry et al., 2007); while recognizing whole words involves large grain-size processes, less affected by attentional manipulation. Results contrasting valid to invalid cueing (e.g., Posner's paradigm) are inconsistent: spatial cueing is sometimes modulated by lexicality (Auclair & Siéroff, 2002), sometimes not (McCann et al., 1992), or is even absent (when stimuli are presented in central vision, Ducrot & Grainger, 2007).

In an attentional paradigm directly relevant to what was done in the current study, Montani et al. (2014) also used a neutral cue that broadens the attention on both left and right spatial locations in addition to (valid and invalid) lateralized cues. A modulation of cueing effects by the lexical status and familiarity of the words was found. Recognition of high-frequency words (HFWs) was *facilitated* by the more global processing induced by the neutral cue, even more than the valid cue, presumably because this broad distribution of attention is the default mode for processing HFWs (Brand-D'abriesca & Lavie, 2007; Ghahghaei et al., 2013; Kliegl et al., 2006). This enhancement of word identification when attention is broadly distributed could explain our finding for greater discrimination responses in the left hemisphere when the task involved deploying visual attention over the two vertical bars to monitor.

Another non-negligible factor lies in the visibility of all letters which is maintained with the deployed attention task, while the central cross could partly mask the central letter (depending on its identity and shape) when using stimuli of 5 letters as done here. This latter condition could thus reduce recognition processes for some of the words and trigger misperception of some of the PW (as words), therefore breaking the periodicity of the words perceived occurrence, and thereby, reducing the selective response at the deviant's frequency.

### 4.4 Individual sensitivity

Assessing the significance of responses at the individual level and the correlations between conditions gave rise to several interesting findings.

First, the high correlation across individuals between responses to words when item repetition versus set size were controlled confirms that the lack of difference observed at the group level is not due to unstable/variable responses. Second, the high co-variation of amplitudes between the two attentional tasks suggests that the greater neural response with the bars task is due to a mere quantitative (deployment of spatial attention) and not a qualitative (e.g., increase in arousal) difference in word processing. This interpretation is reinforced by the lack of correlation between RT increase at the behavioural level between cross and bars tasks and the amplitude increase at the neural level between these two tasks. Finally, correlation was smaller but still significant between word-selective responses when inserted among NW versus PW, suggesting that these two processes of pre-lexical versus lexical access vary similarly across individuals.

Regarding the significance of responses at the individual level, in the coarser contrast (NWW), 100% of participants had at least one significant response for discriminating words, and more than 2/3rd of them (68%) showed significant responses in all four conditions (2 Control Type × 2 Tasks). Results were more mitigated for lexical responses (PWW), where 2 participants did not display any word-response (9%), while about half showed consistent responses in all conditions (45%). Nevertheless, when taking only the LROI (where the response concentrates) and the most sensitive attentional bars task, the rate of significant responses at the lexical level (words in PW) was 82% (18/22 participants), which is impressive. It contrasts with a recent study using English words and pseudowords in a similar design (Barnes et al., 2021) where only a 10% detection rate was reported (testing 10 participants). There may be several reasons for this discrepancy of results. First, we used higher variability than the latter study when generating pseudowords: we kept the syllabic and consonant–vowel structure matched item-wise, but we did not control for letter identity. In (Barnes et al., 2021), items were 4 letter-long, and there was only 1-letter difference between each of the four created PW and their matched words, and each time at the same first position (e.g., “BOOK”, wook, dook, gook, mook). Thus, each word and its 4 matched pseudowords activated the same set of orthographic neighbours in the lexicon. In the orthographic priming literature, unmasked pseudoword neighbour primes have been shown to play an inhibitory role in the word selection process during recognition (Burt, 2009; Carreiras et al., 1997; De Moor & Verguts, 2006; Grainger & Segui, 1990; Versace, 1998). Thus, there might be an impact of repeatedly activating the same orthographic neighbours, acting as competitor items in the lexicon on further recognition processes of target words. Second, language was different. While we tested French-speaking participants, (Barnes et al., 2021) studied English-speaking participants. These two languages vary in several orthographic properties, such as transparency, or the number of orthographic neighbours. English words, for instance, have a greater orthographic neighbourhood size than French words overall, but the difference is even more striking when considering the stimuli used in the two respective experiments: the mean number of orthographic neighbours *N* is 19 for 4-letter English words, while it is 6 only for 5-letters French words (Marian et al., 2012). Neighbourhood size has been shown inconsistently to play a facilitatory (Grainger, 1992; Perea, 2015) or inhibitory (Perea et al., 2008; Pollatsek et al., 1999; Van Heuven et al., 1998; Whitney & Lavidor, 2005) role on word recognition, although the picture is further complicated by both frequency effects and task demands (Grainger & Segui, 1990). In any case, the questions raised by result incongruencies in FPVS studies using words show that it is crucial to design new experiments to understand if the lack of effects observed in (Barnes et al., 2021), in contrast to significant findings here, are due to methodological choices or if language properties as evoked above may play an intrinsic role.

### 4.5 Stimulation frequencies and harmonics

It might seem surprising that the SNR of the word-selective response displayed in Fig. 2 was lower at 2 Hz (the oddball stimulation frequency) and largest at 6 Hz (harmonic of the stimulation frequency). In frequency-tagging, responses of the brain to a (periodic) stimulus may occur at the stimulation frequency F as well as the harmonics of this frequency (Norcia et al., 2015) and the largest response does not always occur at the frequency of stimulation (see e.g., Retter & Rossion, 2016). In ‘oddball’ paradigms as the one we have used here, it is very common to observe the highest SNR at harmonics of the stimulation frequency (i.e., higher than the first harmonic, e.g., Lochy et al., 2015; Volfart et al., 2021). In our opinion, this could be due to two factors at least, that could be further investigated in future research as it was not an objective in the current study. First, the harmonics reflect the shape of the (category-selective) response, which may be better fitted by a 6 Hz sinewave than a 2 Hz sinewave (for a recent review, see Retter et al., 2021); second, since EEG activity belongs to a broad class of physical signals which arise from a so-called 1/f process (e.g., Donoghue et al., 2020; Freeman et al., 2003), it is well established that EEG noise is largest at low frequencies so that responses falling in low frequency ranges – as well as around the alpha band (8–12 Hz) – tend to have a lower SNR. Considering that the SNR values presented in the results are computed relative to the mean amplitude in the neighbouring bins, higher EEG noise in the lower frequencies will inherently be associated with lower SNR values in this same frequency range, relative to harmonics in higher frequency ranges less affected by noise.

## 5 Summary and conclusions

In conclusion, the current study brings several important new findings that help understanding the factors influencing the recorded word-selective EEG responses, thereby supporting the validity and robustness of FPVS studies, with valuable practical applications. They confirm that word-selective responses are linguistic, immune to design-related factors, but may be augmented by using a deployed rather than focal attention task. First, at least when using a relatively large stimulus set, the FPVS-oddball paradigm does measure word-selective responses because of their orthographic or lexical properties and not because of statistical (learning of) (ir)regularities during the stimulation sequences. This is evidenced both by the lack of influence of controlling item repetition, by the LH topography of the response, and by the modulation of the response by the discrimination level (wordlikeness of base stimuli). Second, the orthogonal task performed by our participants has an important impact, with greater discrimination responses when attention is deployed on both sides of the letters-strings rather than being focused on the centre. Third, word-selective neural responses are more clearly identified when the discrimination is coarser (words among nonwords) presumably reflecting a mixture of pre-lexical and lexical processes. In the former case, individual responses in the LROI are significant in 95% of the cases, while they are significant in 82% of the cases when they are discriminated from PW, reinforcing previous findings in French-speaking participants (Lochy et al., 2015).

## Open practices

The study in this article earned Open Data and Open Material Badges for transparent practices. The data and material used in this study are available at https://osf.io/kvfgx/.

## CRediT authorship contribution statement

**Aliette Lochy**: Conceptualization, Methodology, Software, Investigation, Formal analysis, Writing - original draft. **Bruno Rossion**: Conceptualization, Methodology, Funding acquisition, Writing - Review & Editing. **Matthew Lambon Ralph**: Conceptualization, Methodology, Writing - Review & Editing. **Angélique Volfart**: Writing - Review & Editing. **Olaf Hauk**: Writing - Review & Editing. **Christine Schiltz**: Conceptualization, Methodology, Resources, Funding acquisition, Writing - Review & Editing.

## Declaration of competing interest

The authors declare no competing interests.
