# Supplementary material for: Linguistic and attentional factors – Not statistical regularities – Contribute to word-selective neural responses with FPVS-oddball paradigms
Source: Cortex. 2024 Apr;173:339–54. doi: 10.1016/j.cortex.2024.01.007 (PMC10988773; doi:10.1016/j.cortex.2024.01.007)

**SUPPLEMENTARY MATERIAL**

**Linguistic and attentional factors – not statistical regularities – contribute to word-selective neural responses with FPVS-oddball paradigms**

Aliette Lochy^1,2^, Bruno Rossion^3,4^, Matthew Lambon Ralph^5^, Angélique Volfart^6^, Olaf Hauk^5^, & Christine Schiltz^1^

**Table S1: stimuli**. List of 30 experimental words and their consonant-vowel structure, 4 lists of pseudowords (n=120) matched item-wise on CV structure, 4 lists of nonwords (n=120) matched at the letter-level to pseudowords. For mean characteristics at lexical and orthographic level, see text (Table 1). The sets of PW did not differ, as well as the sets of NW, on the mean values for the number of orthographic neighbours and bigram frequency: p-values corresponding to these comparisons are provided below the stimuli.

| Words | CV structure | PW list1 | PW list2 | PW list3 | PW list4 | NW list1 | NW list2 | NW list3 | NW list4 |
| --- | --- | --- | --- | --- | --- | --- | --- | --- | --- |
| chose | CCVCV | crase | chire | brige | glite | rcesa | rcohi | rgebi | tgeli |
| chute | CCVCV | vrile | trute | flabe | glape | rvile | tteru | bfela | lgape |
| drame | CCVCV | plare | trale | pluve | trole | rlaep | rteal | vpelu | lrtoe |
| glace | CCVCV | crure | trove | chule | fraga | rruce | vtore | hlecu | rgafa |
| plume | CCVCV | frabe | trifu | vrale | trise | fbate | rftui | eavlr | rstei |
| chien | CCVVC | trouf | crous | treur | drais | rfuot | uosrc | rrteu | rdsia |
| fleur | CCVVC | crior | flain | claut | frout | rcoir | lniaf | ltuac | uoftr |
| fruit | CCVVC | cleur | chuit | cluin | treul | ucrle | htiuc | lciun | lruet |
| train | CCVVC | chaul | stoux | prait | breux | hluac | txuos | rpiat | rxueb |
| jambe | CVCCV | tulbe | torme | madru | vervi | letbu | teomr | adrmu | ervvi |
| larme | CVCCV | butre | fotte | ravre | covre | etbru | tteof | raerv | rvoce |
| sucre | CVCCV | bivre | lebre | sague | mible | rveib | lrbee | gesae | ibmle |
| tigre | CVCCV | vatre | gacle | varte | tiche | ratve | cglea | aervt | cihte |
| vache | CVCCV | sarve | salpe | dutte | techu | evsra | lsape | tdute | hctue |
| villa | CVCCV | nadre | tivre | berde | roste | adrne | rtiev | eebdr | eosrt |
| canal | CVCVC | bulan | zoler | bacal | tomel | bnual | rzeol | cblaa | mloet |
| divan | CVCVC | vadin | ridan | meton | badon | nvdai | dnira | tmeon | dboan |
| roman | CVCVC | nitar | torin | ledin | nimer | iatrn | ntior | ndile | mrnie |
| sapin | CVCVC | barin | palel | fatil | vucor | rnbia | ealpl | lftai | rvocu |
| genou | CVCVV | rabou | tecou | darai | nague | uaorb | euotc | ridaa | gnaeu |
| radio | CVCVV | virie | pomue | bario | tovui | verii | mpeue | rbaio | vtoiu |
| neige | CVVCV | toudu | bauxe | tiafe | faule | tuudo | xuabe | ftaei | lfuae |
| nuage | CVVCV | naupi | noine | mouse | gouba | npiua | ieonn | smeuo | oagbu |
| piano | CVVCV | vionu | houli | daune | suine | nvoiu | oiulh | dnuea | nseiu |
| arbre | VCCCV | erbra | irdre | onste | inche | eabrr | rrdie | esnto | nhiec |
| oncle | VCCCV | onfle | ancte | onche | ustra | fnloe | acnte | hcnoe | tsrua |
| enfer | VCCVC | erfen | egren | ancal | astin | rfnee | egrne | aanlc | sntai |
| orage | VCVCV | opule | iroge | otire | adite | ueolp | eoirg | rteoi | dtiae |
| usine | VCVCV | orame | ivene | irote | ugone | aomre | vneei | ioetr | uoeng |
| avion | VCVVC | eruit | arous | enait | ocien | rtuei | uraos | itaen | ncioe |
|  |  |  |  |  |  |  |  |  |  |

| Contrast type | ortho. Neighb. | Big.Freq |
| --- | --- | --- |
|  | *p*= | *p*= |
| PW1-2 | 0.947 | 0.884 |
| PW1-3 | 0.947 | 0.878 |
| PW1-4 | 0.956 | 0.888 |
| PW2-3 | 1.000 | 0.979 |
| PW2-4 | 1.000 | 0.988 |
| PW3-4 | 1.000 | 0.987 |
|  |  |  |
| NW1-2 | 0.423 | 0.129 |
| NW1-3 | 1.000 | 0.209 |
| NW1-4 | 0.662 | 0.796 |
| NW2-3 | 0.326 | 0.826 |
| NW2-4 | 0.184 | 0.234 |
| NW3-4 | 0.662 | 0.207 |

**Figure S1. Individual Z-scores for word-selective responses**

| 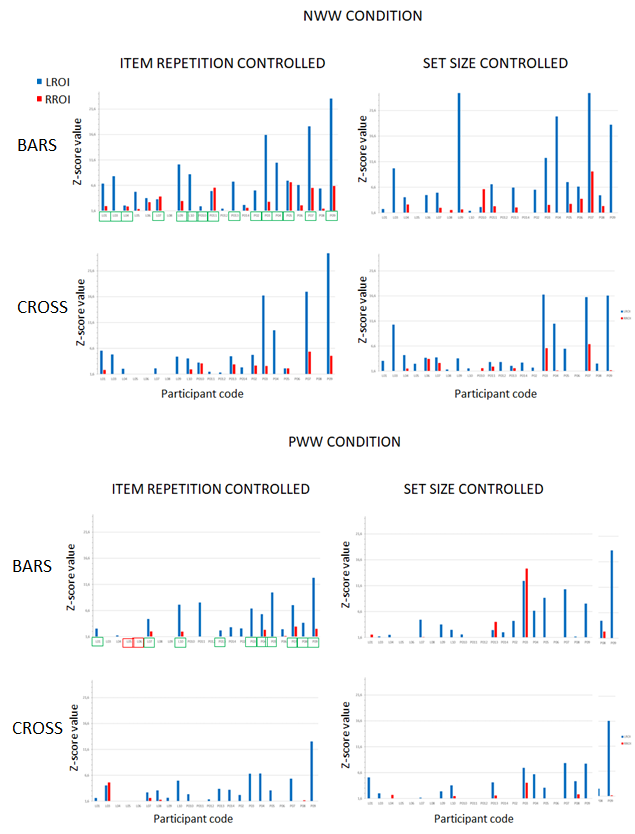 |
| --- |
| Note: **Z-score values for the words discrimination response per participant**, in the LROI (blue) and RROI (red) in each *discrimination level* (Top: coarse level, words among nonwords NWW; Bottom: fine-grained level, words among pseudowords, PWW), each *Control Type* (item repetition, left columns; set size, right columns) and *Tasks* (bars/ cross). Z-scores were calculated on raw amplitude values after computing the sum of 4 harmonics (2Hz – 8Hz) for the discrimination responses. The lowest Y-axis value has been set to *Z*=1.64 (p<.05, 1-tailed). The first top left histogram for each discrimination level indicates the participants who displayed a significant response *in all* tasks and Control Types (green boxes; 15/22 for NWW and 10/22 for PWW) and in *none* (red boxes; 2/22 for PWW). |

**Figure S2. Number of participants with a significant response in the left ROI: A.** Per task x condition (each data point represent 3 minutes testing. In total, there are 76/88 significant data points for NWW, and 58/88 for PWW); **B.** Per task, in *any* condition (at least a significant response in set size *OR* item repetition controlled); **C.** Per task, in *both* conditions (the same individual has a significant response in both set size *AND* item repetition controlled).


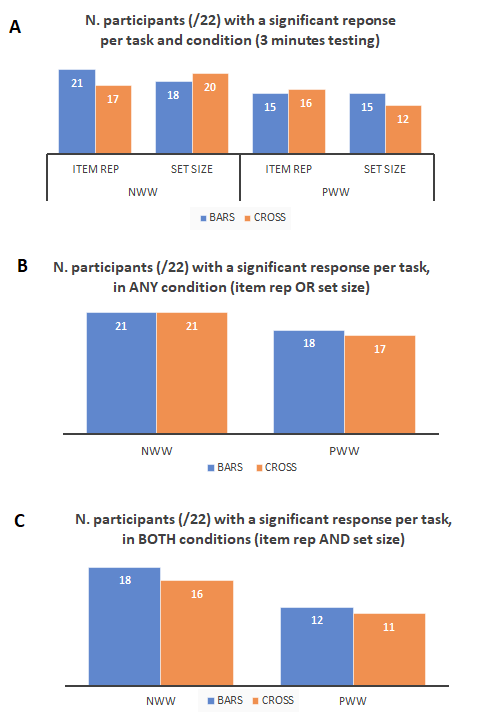

Supplement: Multimedia component 1 [file mmc1.docx]
